# Single-Cell Resolution Imaging of Retinal Ganglion Cell Apoptosis *In Vivo* Using a Cell-Penetrating Caspase-Activatable Peptide Probe

**DOI:** 10.1371/journal.pone.0088855

**Published:** 2014-02-21

**Authors:** Xudong Qiu, James R. Johnson, Bradley S. Wilson, Seth T. Gammon, David Piwnica-Worms, Edward M. Barnett

**Affiliations:** 1 Department of Ophthalmology and Visual Sciences, Washington University School of Medicine, St. Louis, Missouri, United States of America; 2 Molecular Imaging Center, Mallinckrodt Institute of Radiology, BRIGHT Institute, Washington University School of Medicine, St. Louis, Missouri, United States of America; 3 Department of Cancer Systems Imaging, The University of Texas M.D. Anderson Cancer Center, Houston, Texas, United States of America; Schepens Eye Research Institute, Massachusetts Eye and Ear,Harvard Medical School, United States of America

## Abstract

Peptide probes for imaging retinal ganglion cell (RGC) apoptosis consist of a cell-penetrating peptide targeting moiety and a fluorophore-quencher pair flanking an effector caspase consensus sequence. Using *ex vivo* fluorescence imaging, we previously validated the capacity of these probes to identify apoptotic RGCs in cell culture and in an *in vivo* rat model of N-methyl- D-aspartate (NMDA)-induced neurotoxicity. Herein, using TcapQ488, a new probe designed and synthesized for compatibility with clinically-relevant imaging instruments, and real time imaging of a live rat RGC degeneration model, we fully characterized time- and dose-dependent probe activation, signal-to-noise ratios, and probe safety profiles *in vivo*. Adult rats received intravitreal injections of four NMDA concentrations followed by varying TcapQ488 doses. Fluorescence fundus imaging was performed sequentially *in vivo* using a confocal scanning laser ophthalmoscope and individual RGCs displaying activated probe were counted and analyzed. Rats also underwent electroretinography following intravitreal injection of probe. *In vivo* fluorescence fundus imaging revealed distinct single-cell probe activation as an indicator of RGC apoptosis induced by intravitreal NMDA injection that corresponded to the identical cells observed in retinal flat mounts of the same eye. Peak activation of probe *in vivo* was detected 12 hours post probe injection. Detectable fluorescent RGCs increased with increasing NMDA concentration; sensitivity of detection generally increased with increasing TcapQ488 dose until saturating at 0.387 nmol. Electroretinography following intravitreal injections of TcapQ488 showed no significant difference compared with control injections. We optimized the signal-to-noise ratio of a caspase-activatable cell penetrating peptide probe for quantitative non-invasive detection of RGC apoptosis *in vivo*. Full characterization of probe performance in this setting creates an important *in vivo* imaging standard for functional evaluation of future probe analogues and provides a basis for extending this strategy into glaucoma-specific animal models.

## Introduction

Molecular imaging utilizing peptide probes provides a non-invasive means for interrogating cells *in vivo* for a specific intracellular biochemical event [1], [2]. Activatable probes denote such events via enzyme-specific cleavage, resulting in a detectable signal [3], [4]. Through an intramolecular optical quenching strategy, activatable probes with a fluorescent signal moiety remain optically silent until activation by the target enzyme. This probe design confers a high degree of specificity and maximizes the signal-to-noise ratio for optimal detection of events that may occur at a relatively low frequency. This strategy is particularly attractive for application to the eye, as optical imaging can be utilized to identify a fluorescent target using clinically available instruments.

Apoptosis occurs in both normal development and disease in a wide range of tissues and therefore is an attractive target for molecular imaging [5], [6]. Glaucoma, a leading cause of blindness, is characterized by the selective degeneration of retinal ganglion cells (RGC) via apoptosis [7], [8], [9], [10]. Apoptosis can be identified through the activity of the caspase family of proteases, which plays a central role in the enzymatic pathway leading to apoptotic cell death [11], [12]. Previously, we described the design and validation *in vitro* of cell-penetrating peptide probes sensitive to activated effector caspase activity [3], [13], . Intracellular delivery of these molecular imaging probes was enabled through conjugation to a cell-penetrating peptide sequence serving as a targeting moiety to facilitate rapid probe translocation into cells via endocytic pathways [17]. We demonstrated highly specific uptake by RGCs following intravitreal injection of fluorophore conjugated to our modified cell-penetrating peptide sequence [13], and subsequently utilized these probes in a rat model of NMDA-induced RGC degeneration to validate probe activity and localization *ex vivo* [18], [19].

Using the rat NMDA model, herein we fully characterized probe activation *in vivo* using a confocal scanning laser ophthalmoscope (CSLO) and a clinically-relevant protocol. This approach enabled repetitive and sequential non-invasive fluorescence fundus imaging of individual animals to determine the temporal course of probe activation *in vivo*, the optimal kinetics of probe delivery, and a probe dose providing an optimal signal-to-noise ratio in this model. In addition, electroretinogram (ERG) studies were performed to rule out probe-related toxicity. These studies provide a rational basis for future structure-function probe evaluation and for extending these probes and strategies to more disease-specific models of RGC degeneration (i.e., glaucoma), both in rodents and primates.

## Materials and Methods

### Animals

All animal experiments were approved by the Institutional Animal Care and Use Committee at Washington University in St. Louis School of Medicine and adhere to the ARVO Statement for the Use of Animals in Ophthalmic and Vision Research. Male Brown Norway rats weighing approximately 200 g each were purchased from Harlan Laboratories (Indianapolis, IN). All experiments were performed in triplicate or more.

### Activatable Cell-penetrating Peptide (TcapQ488)

TcapQ488 was produced and purified using methods as previously described [17], [18]. This activatable peptide probe consists of an all *D*-amino acid modified Tat cell-penetrating peptide, an *L*-amino acid effector caspase recognition sequence (DEVD), a quencher (QSY7), and a fluorophore (Alexa Fluor-488). This particular fluorophore was chosen for compatibility with clinically-available fluorescent fundus imaging instruments, which are designed for use with either fluorescein or indocyanine green. Upon cleavage of the effector caspase recognition sequence and subsequent loss of fluorescent quenching, fluorescence from the retained intracellular fluorophore is detectable via fluorescence imaging. Stock solutions of purified peptides were formulated in milliQ water at various concentrations and stored at −20°C.

### Intravitreal Injections

All probe, PBS, and NMDA intravitreal injections were performed as described [18], [19]. Briefly, rats were anesthetized by isoflurane inhalation or intraperitoneal injection (0.10 ml/100 g) of a cocktail containing 2 ml ketamine (100 mg/ml), 1.5 ml xylazine (20 mg/ml) and 0.5 ml isotonic sterile saline. Intravitreal injections were performed under a microscope with a U-100 29 gauge, ½ inch length insulin needle and a co-axial 33 gauge, 1 inch length blunt metal needle on a 2.5 µl Hamilton syringe (Hamilton Company, NV). The eye was first punctured by the 29 gauge needle at approximately 1 mm behind the limbus on the nasal side of the eye, aiming toward the optic nerve head. The 33 gauge needle on the 2.5 µl Hamilton syringe was then inserted into the eye through the puncture to deliver injectate. Caution was taken to deliver injectate slowly, over approximately 3 minutes. Erythromycin ointment was applied topically after each intraocular injection.

In experimental groups, eyes were pretreated by injecting 2 µl of 2.5, 12.5, 25 or 40 mM (corresponding to 5, 25, 50 and 80 nmol, respectively) NMDA (Sigma, St. Louis, MO) prepared in 0.01 M phosphate buffered saline (PBS, pH 7.4). Twenty-four hours later, 2.5 µl of 0.039, 0.078, 0.125, 0.155 or 0.310 mM TcapQ488 in PBS were injected into the vitreous. In control experiments, eyes were pretreated with 2 µl of 0.01 M PBS instead of NMDA and followed by 2.5 µl TcapQ488 injection. Animals with visible lens damage, vitreous or retinal hemorrhage or retinal detachment were excluded from analysis, although this was a rare occurrence.

### In Vivo Imaging

**Figure 1:**
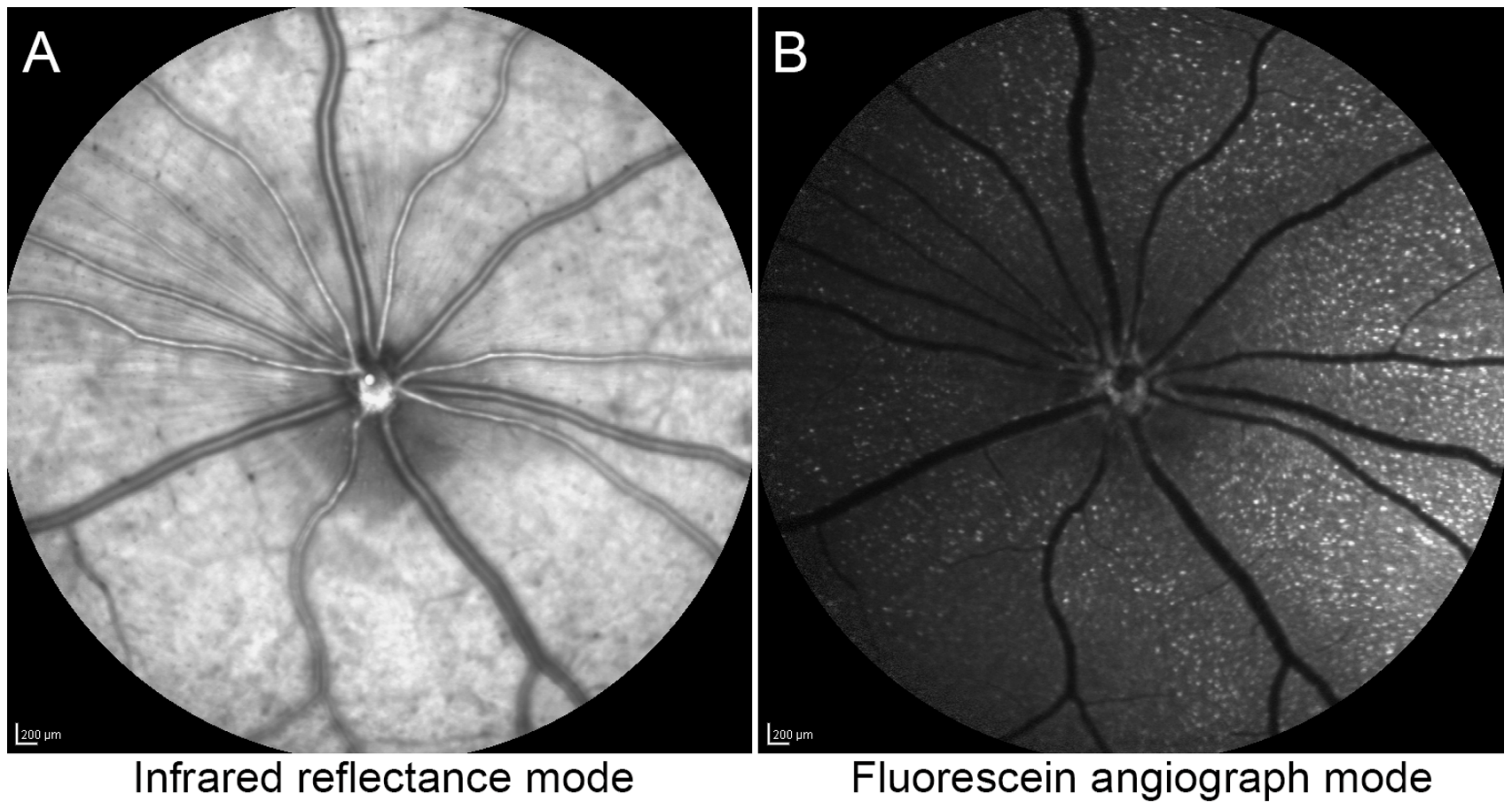
*In vivo* confocal scanning laser ophthalmoscopic imaging of single retinal ganglion cell (RGC) apoptosis. (A) Infrared reflection fundus image at 25 hours post-probe injection from a rat eye pretreated with 25 mM NMDA and followed 24 hrs later by injection of 0.387 nmol TcapQ488. The image was focused on the Nerve Fiber Layer using a 55°FOV lens. (B) Fluorescein angiograph (FA) mode fundus image from the same eye at the same time point and focal plane showing distinct fluorescent signals from probe activation. Both images were recorded as an average of 100 aligned frames to obtain a single low-noise, high contrast image. Scale bar in both images, 200 µm.

A CSLO (Retinal Angiograph II, Heidelberg Engineering, Inc., Germany) equipped with a 55° field of view (FOV) lens was used for *in vivo* image acquisition. Rat retinas were typically imaged at 4, 12, 24, 48 and 72 hours post probe injection. Rats were positioned on a platform attached to the CSLO chin rest. A custom-made PMMA contact lens (Cantor & Nissel Limited, Brackley, UK) was placed on the eye for all imaging to protect the corneal surface and improve image quality. Pupils were dilated with one drop of Tropicamide ophthalmic solution USP 1% (Bausch & Lomb, Tampa, FL). The CSLO image was focused on the retinal nerve fiber layer under infrared reflection mode (IR, diode laser at 820 nm) at 50% intensity to center the optic disc in the image (Figure 1A). Fluorescent angiograph mode (FA, blue solid laser at 488 nm with 500 nm barrier filter) was then used to acquire activated TcapQ488 fluorescence signal of the RGC layer (Figure 1B). All CSLO images were recorded as an average of 100 aligned frames to obtain a single low-noise, high contrast image.

### Ex Vivo Imaging

Retinal flat mounts were prepared and fluorescence microscopy performed as described previously [18].

### Image Analysis and Cell Counting

Images used for data analysis/cell counting were centered at the optic disc. Imaging parameters, including gain, were kept constant during image acquisition. Fluorescence signals were counted manually using Image J software [20] based on fluorescence intensity and sharpness. Manual counting enabled consistent and accurate analysis throughout cell counting. Quantitation by manual counting was confirmed in a subset of animals using “Find Maxima”, an automated cell counting program in ImageJ (http://rsb.info.nih.gov/ij); noise tolerance was set at 17, edge and center (optic disc) maxima were excluded from the analysis field (See Figure S1).

### Electroretinography (ERG) Protocol

Rats were dark-adapted overnight, prepared for recording under dim red illumination, and anesthetized initially by subcutaneous injection of ketamine/xylazine cocktail (0.10 ml/100 g) containing 2 ml ketamine (100 mg/ml), 1.5 ml xylazine (20 mg/ml) and 0.5 ml isotonic sterile saline and supplemented as necessary at 1/4 of the original dose. Pupils were dilated with 1.0% atropine sulfate ophthalmic solution (Bausch & Lomb, Tampa, FL). During testing, a heating pad controlled by a rectal temperature probe maintained body temperature between 36.5°C and 37.5°C. Each rat was positioned with its head just inside the opening of a Model 2503D Ganzfeld stimulator (LKC technologies, Gaithersburg, MD). ERGs were recorded using a platinum loop electrode (2.0 mm in diameter) positioned in a drop of 0.5% atropine sulfate in 1.25% hydroxypropyl methylcellulose (GONAK; Akorn Inc., Buffalo Grove, IL) on the corneal surface of the eye to be tested. A reference needle electrode was inserted under the skin at the vertex of the skull and a ground electrode was inserted under the skin of the back or tail. Flash ERG measurements were performed with a UTAS-E3000 Visual Electrodiagnostic System using EM for Windows software (LKC Technologies, Inc., Gaithersburg, MD). The stimulus (trial) consisted of a brief full field flash (10 µs), either in darkness or in the presence of dim (29 cd/mm) or bright (198 cd/mm) background illumination after 10 minutes of light adaptation. The initiation of the flash was taken as time zero. The response was recorded over 250 ms plus 25 ms of pre-trial baseline. Responses from several trials were averaged and the interval between flash trials was adjusted so that responses did not decrease in amplitude over the trial series of for each step. The log light intensity (log [^cd*s^/_m2_]) was calculated based on the manufacturer’s calibrations. To reduce noise interference, a 60 Hz digital notch filter was used for weak responses (routinely for intensities below −2 log; as needed for brighter intensities).

Rats underwent ERG testing pre-injection, at 1 week and at 2 months post intravitreal injection of TcapQ488. Probe concentrations of 0.078 mM and 0.155 mM in 2.5 µl (0.193 and 0.387 nmol, respectively) were examined. Control rats underwent PBS injection only. All conditions were tested in triplicate.

### Statistical Analyses

Data were analyzed using SAS version 9.3 (Base SAS® 9.3, Procedures Guide: Statistical Procedures, SAS Institute, Inc., Cary, NC, 2011). All p-values were two-tailed. A p-value <0.05 was considered significant. An analysis of variance was performed on the log transformed probe activation data to determine the effect of eye, light intensity, probe dose, and NMDA concentration on probe activation. Simple effects tests were done to determine significant effects of probe dose at each NMDA concentration and vice versa. For ERG data, a repeated measures analysis of variance was used to determine the effect of eye, probe, and time on B-wave amplitude. Significant interactions were followed-up with simple effects tests for probe conditions and time periods. Significant simple effects tests were further investigated using pair-wise comparisons.

## Results

### Correspondence of *In Vivo* and *Ex Vivo* Imaging of Probe Activation

**Figure 2:**
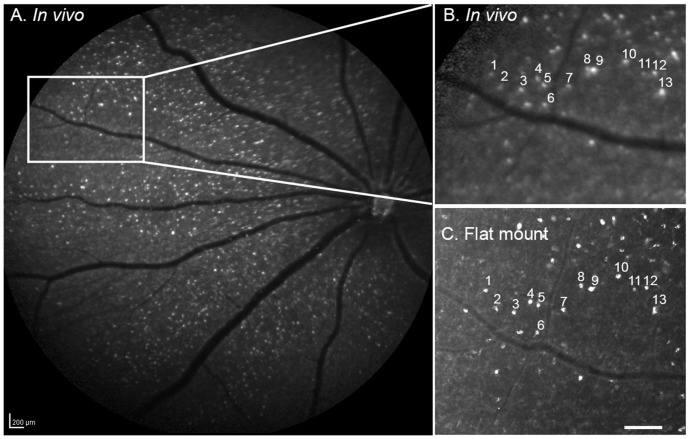
Correspondence of an *in vivo* fluorescent fundus image with an *ex vivo* retinal flat mount. (A) Fluorescent fundus image obtained *in vivo* using the CSLO (28 hours post-probe injection) from a rat eye pretreated with NMDA followed by TcapQ488. Strong, punctate fluorescent signals were detected in the retina ganglion cell (RGC) layer. (B) Higher magnification of the boxed area in A in which prominent fluorescent signals are highlighted. (C) *Ex vivo* flat mount of the same retina showed excellent correspondence with *in vivo* images in A and B, indicating that real time images reflect single cell resolution of probe activation. Scale bar: A, 200 µm; C, 100 µm.

Strong fluorescent signals were observed with *in vivo* imaging in the RGC layer of eyes of living rats pretreated with NMDA followed by TcapQ488 (Figure 1B). The fluorescence was cell-specific and punctate, consistent with our previous *ex vivo* observations that fluorescence was restricted to the cell bodies of individual RGCs containing activated probe. Several eyes underwent *in vivo* imaging followed by histological assessment of retina flat mounts *ex vivo* to directly compare *in vivo* fluorescence fundus images with *ex vivo* flat mount fluorescence microscopy images of the same retina. Retinal flat mounts displayed distinct fluorescent signals in individual cell bodies, consistent with probe activation in a specific subset of cells. Comparison of *in vivo* and *ex vivo* images revealed excellent correspondence of the same individual fluorescent cell bodies (Figure 2). As documented previously [18], vertical retinal sections confirmed that the vast majority of fluorescent cell bodies, with the exception of a few cells in the inner nuclear layer, were located within the RGC layer (data not shown). Spread of fluorescence to the optic nerve suggested that at least the cleavage product, in this case Alexa Fluor 488, may enter axonal transport pathways in RGCs.

### Probe Activation as a Function of Time

**Figure 3:**
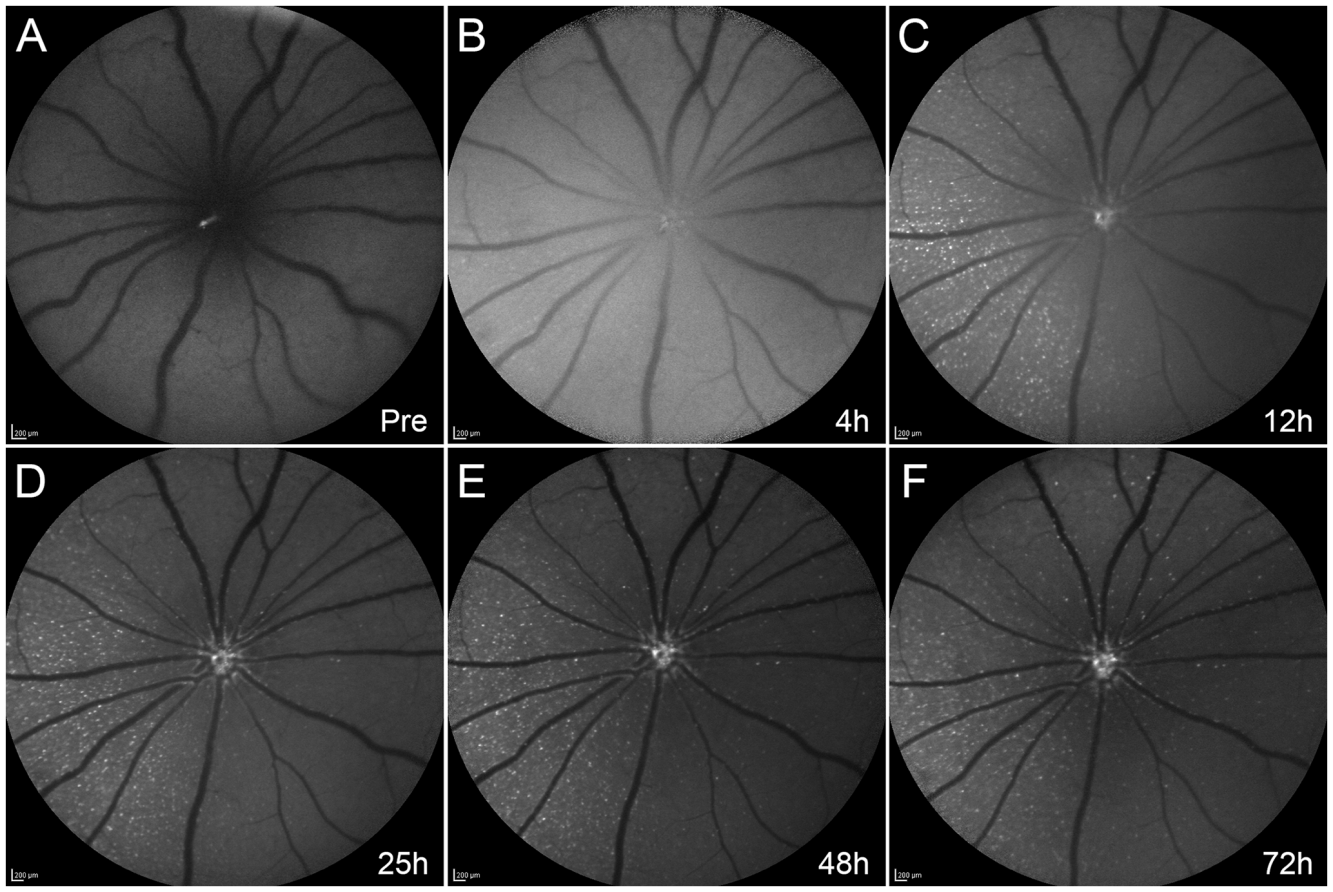
Representative kinetics of TcapQ488 activation *in vivo*. *In vivo* images were taken in a rat eye pretreated with 12.5 mM NMDA immediately before (A) and at 4 hours (B), 12 hours (C), 25 hours (D), 48 hours (E) and 72 hours (F) post intravitreal injection of 0.313 nmol TcapQ488. Evidence of initial probe activation was noted at 4 hours after TcapQ488 injection (B). Scale bar, 200 µm in all images.

**Figure 4:**
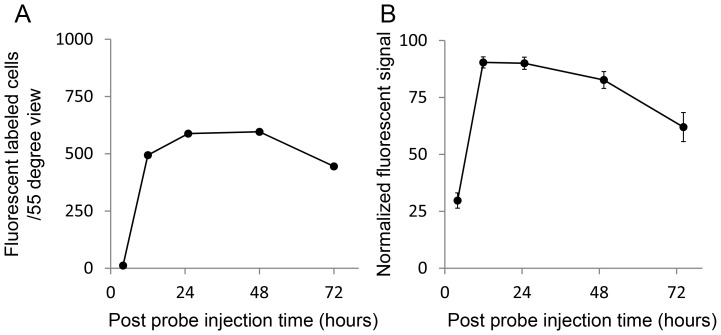
Quantitative plot of probe activation from live images. (A) Plot of probe activation from images in Figure 3 B–F. (B) Probe activation normalized to maximum counts from an individual eye at each time point was averaged across all probe dose/NMDA concentration combinations. Probe activation increased significantly in the first 12 hours and generally decreased slowly thereafter. Data represent mean ± SEM.

Following pre-treatment with NMDA and TcapQ488 injection, serial *in vivo* fluorescence fundus imaging in individual animals using the CSLO enabled clear delineation of the spatial and temporal pattern of probe activation (Figures 3, 4). Animals were routinely imaged immediately before probe injection and five times post-probe injection (at 4, 12, 24, 48 and 72 hours) to determine the kinetics of probe activation *in vivo* and the optimal time point for detecting probe activation. TcapQ488 activation was evident 4 hours post probe injection (Figures 3, 4). Notably, stronger fluorescent signal was noted near the injection site, consistent with a subtle gradient effect reflecting diffusion of both probe and NMDA throughout the vitreous from the injection site. Figure 4A shows the time course of fluorescent probe activation determined from analysis of the images from Figure 3B–F. Probe activation increased significantly in the first 12 hours, remaining relatively steady out to 72 hours. As expected, probe activation signals were dependent on probe dose and NMDA concentration, but this kinetic pattern was remarkably consistent across all probe dose and NMDA concentration combinations (). Figure 4B represents a normalized analysis of probe activation kinetics over the first 72 hours based on pooled data across the study. From this kinetic analysis, the 12 hour time point was utilized to quantify probe activation across all NMDA and probe concentrations.

### Probe Activation as a Function of NMDA Concentration

**Figure 5:**
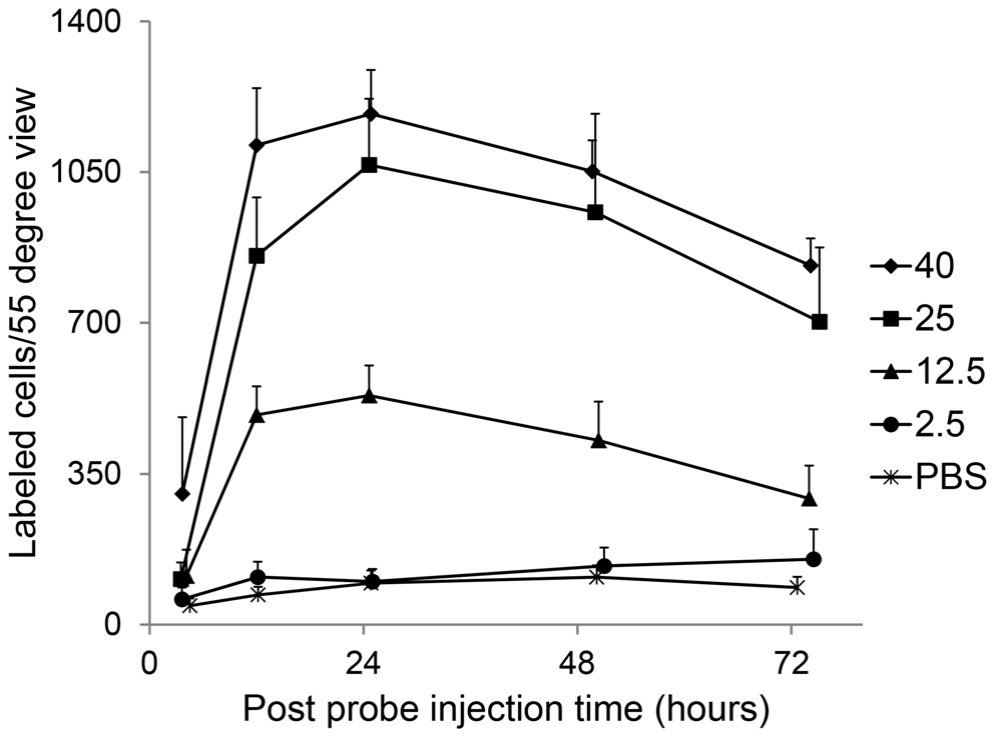
*In vivo* probe activation as a function of NMDA concentration. Rat eyes were pretreated with various NMDA concentrations (2.5, 12.5, 25, 40 mM) followed by 0.313 nmol TcapQ488. A control injection consisting of PBS was also performed to determine background probe activation levels in the absence of NMDA. All eyes were imaged at 4, 12, 24, 48 and 72 hours post-probe injection. Probe activation increased with increasing NMDA concentration at all time points examined. n≥3 (5 to 8 eyes) for each time point. Data represent mean ± SEM.

**Figure 6:**
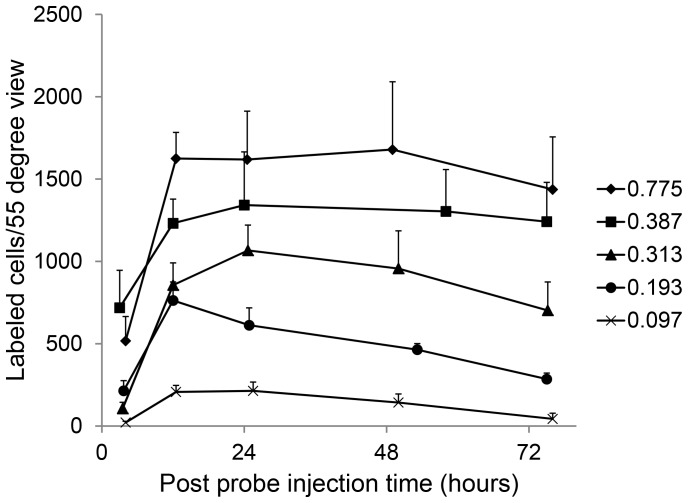
*In vivo* probe signal as a function of TcapQ488 dose. Rat eyes were pretreated with 25(0.097, 0.193, 0.313, 0.387 and 0.775 nmol). All eyes were imaged at 4, 12, 24, 48 and 72 hours post-probe injection. Probe activation increased with increasing TcapQ488 doses at all but the earliest time point (4 hours post probe injection). n≥3 (5 to 8 eyes) for each time point. Data represent mean ± SEM.

**Figure 7:**
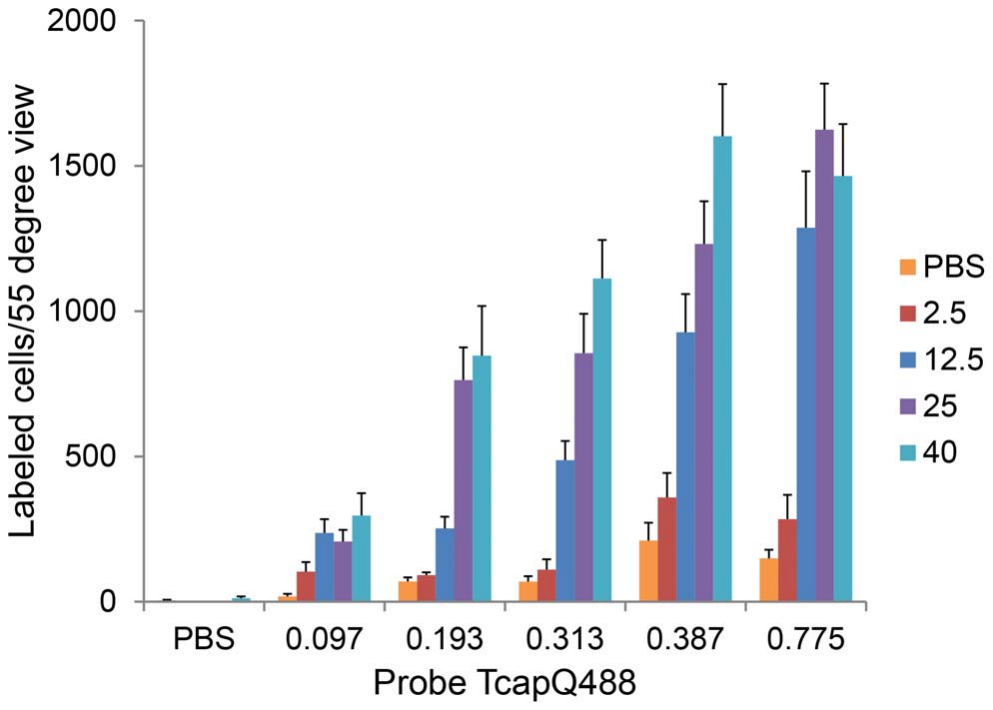
Probe activation *in vivo* as a function of both NMDA concentration and probe dose. Retinal ganglion cell apoptosis was induced in rats by intravitreal injection of NMDA at various concentrations (0 (PBS only), 2.5, 12.5, 25 and 40 mM), followed by intravitreal injection of TcapQ488 (at 0 (PBS only), 0.097, 0.193, 0.313, 0.387 and 0.775 nmol) for a total of 27 NMDA-probe-dose combinations. Data reflect imaging at 12 hours post-probe injection. Probe signal increased as both NMDA concentration and probe dose increased. The plateau in TcapQ488 activation above 0.313 nmol indicates saturation of the dynamic range of probe dosing. n≥3 (5 to 8 eyes) at each combination. Data represent mean ± SEM.

We previously determined the frequency of probe activation at one single time point across multiple NMDA concentrations through *ex vivo* analyses of retinal flat mounts [18]. Using the same model and NMDA concentrations (2.5, 12.5, 25, 40 mM) with *in vivo* imaging, we confirmed the NMDA concentration-response relationship with probe activation at multiple time points post-probe injection (Figure 5). Control injections of PBS were also performed to determine the background level of probe activation in the absence of NMDA excitotoxicity. As expected, NMDA-induced apoptosis, indicated by TcapQ488 activation in individual RGCs, occurred in a dose-dependent manner as documented by *in vivo* imaging. Significant interaction between probe and NMDA was observed (p = 0.015) indicating that probe activation depended on NMDA concentration. There were significant differences in probe activation among the four NMDA concentrations across all probe doses (TcapQ488 dose (nmol): 0.097, p = 0.0001; 0.193, p<0.0001; 0.313, p<0.0001; 0.387, p = 0.0064; 0.775, p<0.0001), but not for PBS (PBS, p = 0.45). Increased probe activation was observed with greater NMDA concentrations at nearly all probe doses examined (Figures 6, 7), the exception being at 0.097 nmol TcapQ488, where probe activation frequency was relatively small for all four NMDA concentrations (pair-wise tests not performed) (Figure 7). This was documented at all 5 time points examined (4, 12, 24, 48 and 72 hours post-probe injection), again with maximal or near-maximal TcapQ488 activation observed at 12 hours post-probe injection (Figures 5, S2).

### Probe Signal as a Function of Probe Dose

To determine the probe concentration that maximized RGC apoptosis detection while minimizing background probe signal, five TcapQ488 doses (0.097, 0.193, 0.313, 0.387 and 0.775 nmol) were examined at each standard NMDA concentration (Figures 6, 7, S2). TcapQ488 activation across probe doses for 25 mM NMDA is shown in detail in Figure 6. As TcapQ488 dosage increased, the probe activation frequency also increased, suggesting that the sensitivity of TcapQ488 in detecting apoptosis using our *in vivo* imaging strategy was dependent on probe dose. Figure 7 shows probe activation results for each combination of NMDA concentration and TcapQ488 dosage at the 12 hour time point. There were significant differences in activation frequency across probe doses at all NMDA concentrations (2.5 mM, p = 0.02; 12.5 mM, p = 0.002; 25 mM, p<0.001; 40 mM, p<0.001). Probe activation generally increased as probe dose increased for each NMDA concentration. The relationship between probe dose and activation, however, began to saturate at higher probe doses indicating that further increases would likely be of limited benefit (Figure 7). Consistent with this, pair-wise tests revealed no significant difference in probe activation between probe doses of 0.387 nmol and 0.775 nmol at all NMDA concentrations. In addition, pair-wise comparisons of consecutive probe doses higher than 0.193 nmol did not reach statistical significance at any NMDA level with the exception of 0.313 nmol versus 0.387 nmol at 2.5 mM NMDA (p = 0.016). Figure S2 shows a comprehensive 3-dimensional plot of probe activation as a function of NMDA concentration and probe dose at all 5 time points.

The frequency of “background” probe activation, as indicated by PBS-only control injections, also gradually increased with increasing probe doses and there was a significant difference found across probe doses in the PBS condition (p<0.0001). This relationship was not as linear as that seen at each NMDA concentration (Figure 7). Instead, probe activation frequency in the PBS condition remained relatively low until probe dose was increased from 0.313 nmol to 0.387 nmol with pair-wise testing revealing a significant increase (p = 0.013).

**Figure 8:**
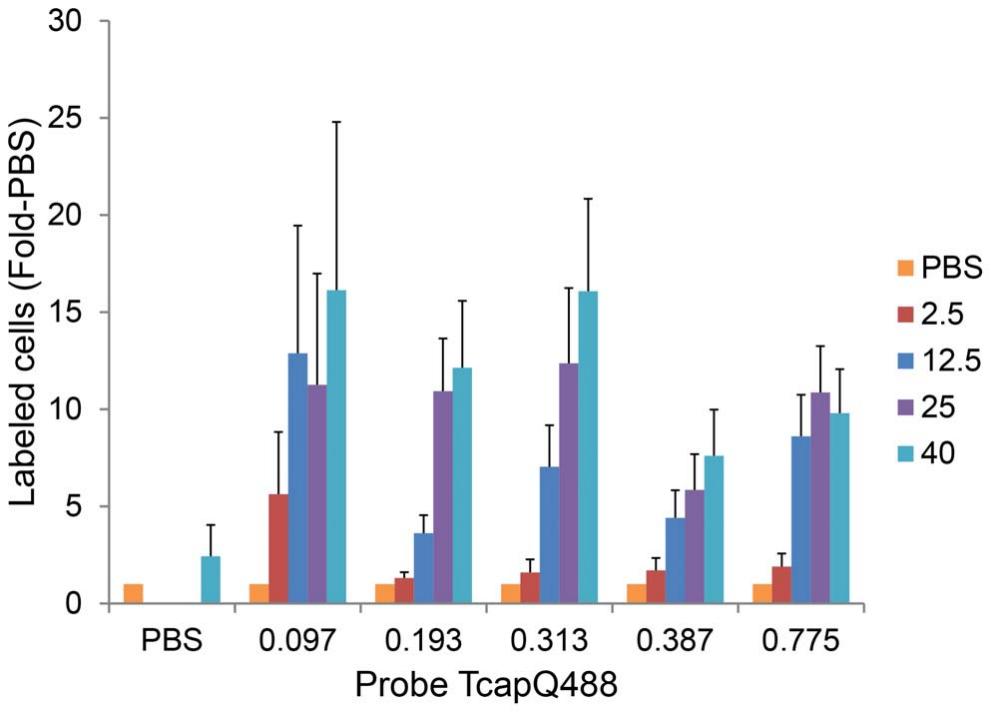
Signal-to-noise ratio across NMDA concentrations and TcapQ488 probe doses. TcapQ488 activation data from Figure 7 was normalized to PBS pre-treatment (i.e., NMDA = 0) for each probe dose to reflect relative “signal-to-noise.” For lower NMDA concentrations (2.5 and 12.5 mM), the ratio was highest at 0.097 nmol probe, while for higher NMDA concentrations (25 and 40 mM), the ratio was similar from probe doses 0.097 to 0.313 nmol. Data represent mean normalized labeled cells ± Error Propagation.

To better evaluate which probe concentration resulted in the best signal-to-noise ratio at the NMDA concentrations examined, data from Figure 7 were reanalyzed as *fold-increase over PBS* across NMDA and TcapQ488 dose combinations at 12 hours post-probe injection (Figure 8). Consistent with the findings that TcapQ488 activation began to saturate at probe doses higher than 0.313 nmol and “background” probe activation also increased significantly at doses higher than 0.313 nmol, a probe dose of 0.313 nmol exhibited the best signal-to-noise ratio, linearity, and the largest dynamic range across NMDA concentrations. For lower NMDA concentrations (2.5 and 12.5 mM), which resulted in less frequent probe activation, the signal-to-noise ratio was optimized at the lowest probe dose examined (0.097 nmol), but the overall dynamic range was reduced.

### ERG Assessment for Toxicity

**Figure 9:**
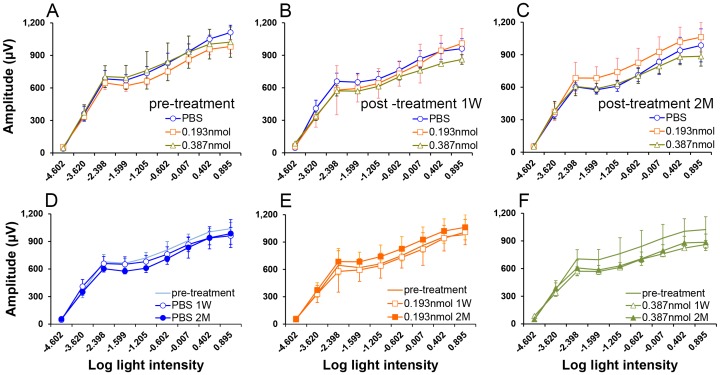
Dark-adapted B-wave amplitudes as a function of probe dose and time post-treatment. Treatment indicates PBS or probe only injections. (A–C) Comparison among pre-treatment, 1 week post-treatment, and 2 months post-treatment time points for each probe condition (PBS, 0.193 nmol TcapQ488, 0.387 nmol TcapQ488). (D–F) Comparison of each probe condition at each time point (pre-treatment, 1 week and 2 months post-treatment). There were no significant differences on dark-adapted B-wave amplitudes among probe conditions when tested at each time point. Data represent mean ± SD.

ERG analysis consisted of dark-adapted A-wave amplitudes, dark- and light-adapted B-wave amplitudes, and implicit time measurements. We performed ERG measurements on eyes receiving intravitreal injections of PBS or TcapQ488 to assess for possible retinal toxicity related to probe injection. Since rods are the dominant photoreceptor subtype in rodents [21], only dark-adapted B-wave data are presented (Figure 9). Comparison between pre-injection, 1 week post-injection, and 2 months post-injection was performed for each probe condition (PBS, 0.193 nmol TcapQ488, 0.387 nmol TcapQ488) (Figure 9A–C). Statistical analysis of mean B-wave amplitudes revealed a significant interaction between the three probe conditions and three time points (p<0.001). There were no significant differences, however, among probe conditions when tested at each time point (pre-injection, p = 0.63; 7 days, p = 0.27; 2 months, p = 0.11). The dark-adapted B-wave showed a mild diminution in amplitude for each condition at 1 week compared with pre-injection, which reached significance for PBS (p = 0.010) and 0.387 nmol probe (p<0.0001), but not for 0.193 nmol probe (p = 0.35). This decrease continued to be statistically significant at 2 months for both PBS (p<0.0001) and 0.387 nmol probe (p<0.0001), while 0.193 nmol probe actually showed a significant increase versus pre-injection (p = 0.004) (Figure 9E). Taken together, these data were consistent with minimal outer retinal toxicity as a function of probe concentration, particularly as the effect appears attributable to the intravitreal injection itself (PBS) rather than probe exposure.

## Discussion

Currently, there are several strategies for diagnosing and monitoring human glaucoma, including intraocular pressure measurement, perimetric assessment of the visual field, examination of the optic disc, and assessment of the optic disc and nerve fiber layer by clinically available imaging devices. These approaches for assessing RGCs are limited primarily to identifying RGC loss and subsequent changes to the field of vision, optic disc, or nerve fiber layer and therefore fail to provide real-time information regarding the status of RGCs which may reside in early injury or pre-death states. Thus, there is a need to improve our ability to monitor the course of RGC injury in glaucoma to assess therapy over time with a more facile marker of disease progression. Molecular imaging with activatable probes provides one such method and has potential to improve our ability to detect and monitor apoptotic processes in RGCs over time, guiding therapeutic choices in the management of this disease. The ability to monitor RGC status without the delays inherent in current clinical approaches should serve to reduce the duration and expense of glaucoma clinical trials and may accelerate the evaluation of potential neuroprotective agents in both animal models and human trials.

Use of a cell-penetrating activatable probe for *in vivo* imaging of RGC physiology has not been previously reported. Herein, TcapQ488 detects effector caspase activity signifying apoptotic cascade activation within a select subset of RGCs in rat retina. These results confirm our previous *ex vivo* analysis of retinal flat mounts using the same NMDA model in rats [18]. Direct comparison of images *in vivo* and flat mounts *ex vivo* from the same animal showed precise correlation of fluorescence in individual RGCs, confirming that the focal fluorescence seen on *in vivo* imaging faithfully represents our previous findings. Also as noted previously, the frequency of cells displaying probe activation *in vivo* showed the expected dose-response relationship with NMDA. This finding was now confirmed across multiple probe doses and was consistent at each concentration studied.

*In vivo* imaging enables repeated analysis of probe activation in a single animal across multiple time points post-intravitreal injection. Using this strategy, we identified a representative time point, 12 hours post injection, at which maximal or near maximal probe activation occurred. This time point was consistent across probe and NMDA concentrations and was therefore used for most analyses. Additional analyses will be required to determine whether the same cells showed evidence of probe activation throughout the first 72 hours post-probe injection, or whether the totals represent a flux between ongoing probe activation and eventual loss of fluorescence in individual RGCs as apoptosis progresses to completion. The half-life of unactivated probe and activated probe fluorescence, and the possibility of uptake by phagocytic cells also require additional study. Given that our approach results in distinct single-cell fluorescence, it should be possible in future studies to focus on the fate of individual cells throughout the duration of fluorescence resulting from probe activation. This will be critical to understanding whether probe activation will persist in the presence of a repeated or ongoing insult, as might occur with elevated intraocular pressure, or whether repeated injections of probe might be informative.

Rigorous 3-dimensional titration curves of probe activation were generated based on NMDA concentration, TcapQ488 dosage, and time. This analysis was needed to determine probe concentrations which would maximize the signal-to-noise ratio, which is critically important for eventual application of this strategy in human glaucoma. The use of the lowest probe concentration possible should reduce the risk, if any, of potential probe-related retinal toxicity. As anticipated, detection sensitivity increased with increasing probe dose, but only to a point. Increasing TcapQ488 dosage above 0.313 nmol provided minimal additional benefits in terms of sensitivity and may increase any potential probe-related toxicity. Our signal-to-noise analysis indicated by the ratio of fluorescent labeled cells at each NMDA and probe dose to that seen in the PBS-only condition, varied with each of these parameters. As discussed previously [22], the relative frequency of RGC apoptosis is expected to be much less in human glaucoma than that observed at the higher concentrations of NMDA utilized in this analysis. As such, the sensitivity to detect apoptosis at a relatively low concentration of NMDA (i.e., 5 nmol) may be of greater importance than that seen at higher concentrations. In our analysis, the signal-to-noise ratio was maximized for the two lowest NMDA conditions at a much lower probe dose. This suggests that for a low apoptosis frequency, the decreased “noise” offered by a lower dose of probe outweighs the benefits of increased sensitivity and signal amplification seen at higher doses.

One distinct advantage of an activatable peptide probe is that activation and subsequent fluorescence signal should not occur until intracellular uptake and cleavage by the specific target enzyme has been initiated. Ideally there should be no probe activation in the PBS only condition. However, evidence of probe activation was detected in the absence of NMDA at the higher probe doses. Several possible mechanisms apply. TcapQ488 activation could reflect a baseline level of RGC apoptosis which is occurring in the rat retina, although this would be expected to be relatively infrequent. Probe activation could result from intravitreal injection-related RGC apoptosis regardless of the nature of the injectate, being related to the small increase in intraocular pressure resulting from even the small volumes (2 µl) utilized in this study [23]. In either case, the frequency of probe activation in the PBS-only condition would be expected to be stable across all probe concentrations examined. Since there was a clear increase in probe activation in the PBS-only condition going from 0.313 nmol probe to 0.387 nmol, these two factors would not fully explain our findings. Probe-associated fluorescence could occur through a concentration-dependent non-specific dequenching of fluorescence not involving true probe activation. Probe activation could also result from probe-related toxicity causing apoptosis in a subset of RGCs, resulting in probe activation. Assuming the vitreous volume of an adult rat eye to be ∼56 µl [24] and the probe volume for the 0.313 nmol dose, the effective concentration of probe in the vitreous was ∼ 5.5 µM. While below the previously determined LD_50_ of 10±2 µM from *in vitro* experiments with a similar probe [3], a subset of cells may show toxicity at this dose. Either mechanism could account for the fact that probe activation under the PBS-only condition was at least partly probe dose-dependent. Alternatively, the “background” probe activation might reflect a combination of both probe dose-dependent and -independent effects.

To move our probe toward future clinical usage, physiological evidence of potential probe toxicity was assayed by electroretinogram (ERG). Full-field ERG has been used in both humans and lower animals as an indicator of retinal function and ERG changes can provide evidence of retinal injury or toxicity, particularly to the outer retina [25], [26]. Under conditions of dark adaptation, the A-wave is generally associated with rod photoreceptor activity while the B-wave is associated with a combination of Muller and bipolar cell layer activity [27]. While these results do not specifically address RGC or inner retinal toxicity, they do provide a means for assessing potential overall retinal toxicity related to the intravitreal probe injection, particularly at higher probe doses. Overall, there was little evidence in our ERG analyses to suggest probe toxicity. Maximal signal-to-noise ratios and linearity occurred at 0.313 nmol of probe with minor toxicity observed both by potential autoactivation and B-wave imaging, but 0.193 nmol still yield nearly identical signal-to-noise ratios (Figure 8), minimal autoactivation, and no observable toxicity via B-wave imaging.

Several molecular strategies for apoptosis detection have been developed. One approach exploits the high affinity of annexin V for phosphatidylserine [28]. This phospholipid is located primarily in the inner leaflet of the plasma membrane of normal cells and is externalized to the outer leaflet early in the commitment to apoptosis [29]. Optical imaging with annexin V-Alexa Fluor 488 has been applied to the retina [30], [31]. While of considerable promise, a fundamental criticism of imaging with annexin V is that the polypeptide binds to phosphatidylserine regardless of whether the phospholipid is exposed on the external leaflet or, if accessible, on the internal leaflet. Cells that undergo necrosis develop defects in the plasma membrane that enable large molecular weight molecules like annexin V access to phosphatidylserine residing in the inner leaflet and thus, in the absence of a second marker of membrane integrity (e.g., propidium iodide), these labeled polypeptides do not distinguish apoptosis from necrosis. This limits diagnostic specificity of the technique by any imaging modality. Other limiting factors for the general use of annexin V include rapid serum half-life of the polypeptide, poor penetration of the blood-brain barrier (and the blood-retinal barrier), pharmacokinetic complexities related to size, potential for immunogenicity, and low signal-to-noise ratios for imaging applications [32], [33], [34].

Modular probe design as utilized in our approach represents a distinct advantage. Any or all probe components – targeting sequence, quencher, fluorophore, and cleavage site – may be altered to achieve a desired result. Given the preferential uptake by RGCs of our cell-penetrating peptides as previously demonstrated [13] and the potential to conjugate them to diverse moieties [17], may render these CPPs useful for delivering other molecular imaging probes to RGCs. Alternatively, further modifications to these CPPs may result in probes with enhanced uptake by other retinal cell types. While identification of CPPs which cross the blood-retinal barrier is desirable, intravitreal injection of retinal therapeutics has become increasingly commonplace in recent years with a low incidence of adverse events [35] Similarly, while TcapQ probes are designed to detect enzymes integral to the apoptotic cascade, it is clear that this platform will accommodate specific peptide sequences recognized by other proteases, kinases, and enzyme activities. As such, our current “proof of concept” experiments should provide the basis for a broad class of novel imaging agents with applications throughout ophthalmology. These applications are not limited to imaging, as “theranostic” probes may ultimately be designed with both imaging and therapeutic capabilities. Identification of important structure-function relationships is an ongoing process facilitated by our ability to evaluate next generation probes in both well-established *in vitro* [14], [16], [19] and *in vivo* (NMDA) models.

The NMDA model employed here is ideal for both the initial validation and future structure-function analysis of these molecular imaging probes. The model is well-established, titratable, highly reproducible, has a relatively short turnover time, and apoptosis can be largely limited to RGCs. As we move towards further pre-clinical validation of this and future generation probes, a more disease-specific animal model of RGC degeneration resulting from elevated intraocular pressure and more closely mimicking human glaucoma in terms of the time course and frequency of RGC apoptosis is desirable. Several such models exist in rodents and primates and will provide a means for further translation of this approach towards human application [36]. Our current results will guide our choice of probe concentration and imaging intervals going forward into more advanced glaucoma-specific models.

## Supporting Information

Figure S1 **Comparison of manual fluorescent RGC cell counting versus automated cell enumeration using ImageJ.** Images at 4, 12, 24, 48, and 72 hours post probe injection were analyzed by manually counting fluorescent cells using Image J software. Fluorescent cell counts were based on fluorescence intensity and sharpness in a group of animals (n = 6) treated with 25 nmol NMDA and 0.313 nmol TcapQ488. The same images were analyzed using the automated counting program “Find Maxima” in ImageJ (http://rsb.info.nih.gov/ij). Noise tolerance was set to 17, edge and center (optic disc) maxima were excluded from the analysis field. Fluorescent cell numbers derived from each method are plotted as a function of probe injection time. Data represent mean ± SD.
(TIF)

Figure S2 **Comprehensive 3-dimensional plot of probe activation** ***in vivo*** **as a function of NMDA concentration and probe dose at five time points post-injection.**
(TIF)
