# Supplementary figures and images for: Single-Cell Resolution Imaging of Retinal Ganglion Cell Apoptosis In Vivo Using a Cell-Penetrating Caspase-Activatable Peptide Probe
Source: PLoS One. 2014 Feb 21;9(2):e88855. doi: 10.1371/journal.pone.0088855 (PMC3931650; doi:10.1371/journal.pone.0088855)

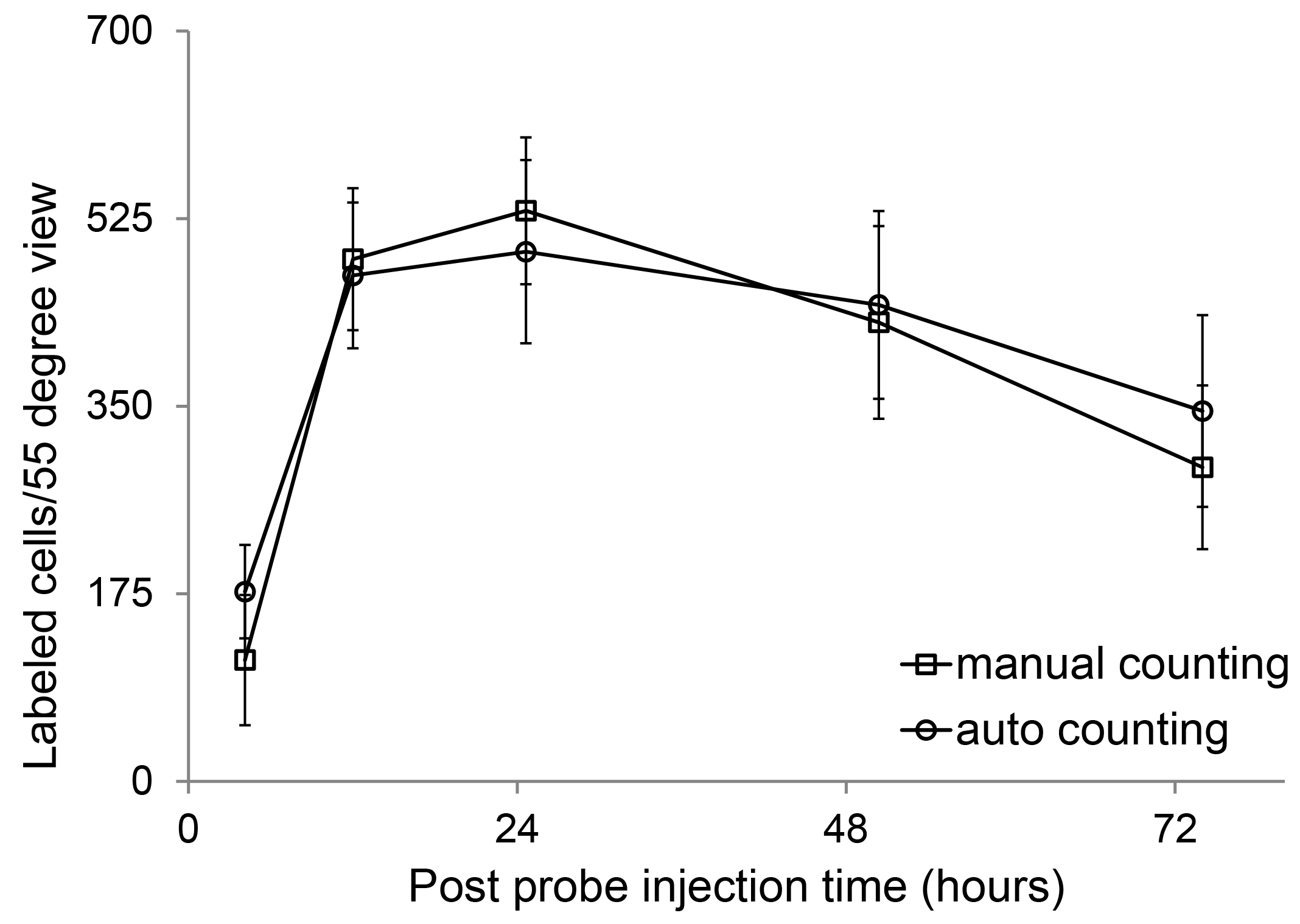

Supplement: Figure S1 — Comparison of manual fluorescent RGC cell counting versus automated cell enumeration using ImageJ. Images at 4, 12, 24, 48, and 72 hours post probe injection were analyzed by manually counting fluorescent cells using Image J software. Fluorescent cell counts were based on fluorescence intensity and sharpness in a group of animals (n = 6) treated with 25 nmol NMDA and 0.313 nmol TcapQ488. The same images were analyzed using the automated counting program “Find Maxima” in ImageJ (http://rsb.info.nih.gov/ij). Noise tolerance was set to 17, edge and center (optic disc) maxima were excluded from the analysis field. Fluorescent cell numbers derived from each method are plotted as a function of probe injection time. Data represent mean ± SD. (TIF) [file pone.0088855.s001.tif]

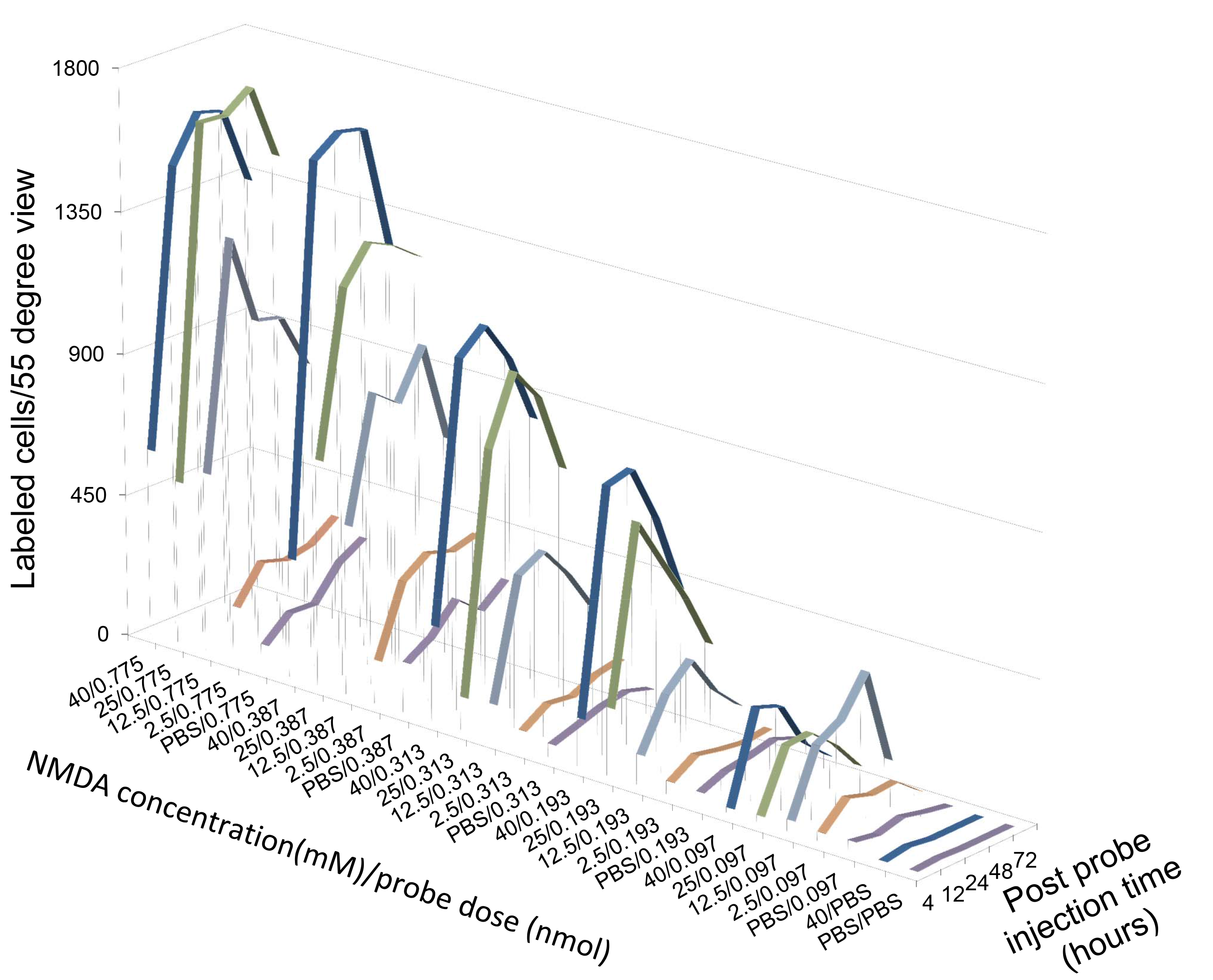

Supplement: Figure S2 — Comprehensive 3-dimensional plot of probe activation in vivo as a function of NMDA concentration and probe dose at five time points post-injection. (TIF) [file pone.0088855.s002.tif]
